# Association of Positive Airway Pressure Adherence with Clinical Outcomes in Patients with Type 2 Diabetes and Obstructive Sleep Apnea

**DOI:** 10.3390/diagnostics14242781

**Published:** 2024-12-11

**Authors:** Izolde Bouloukaki, George Stathakis, Violeta Moniaki, Eleni Mavroudi, Ioanna Tsiligianni, Sophia Schiza

**Affiliations:** 1 Sleep Disorders Center, Department of Respiratory Medicine, School of Medicine, University of Crete, 71410 Heraklion, Greece; stathakisgg@gmail.com (G.S.); vmoniaki@yahoo.gr (V.M.); elenima23@hotmail.com (E.M.); schizas@uoc.gr (S.S.); 2 Department of Social Medicine, School of Medicine, University of Crete, 71410 Heraklion, Greece; i.tsiligianni@uoc.gr

**Keywords:** obstructive sleep apnea, type 2 diabetes mellitus, positive airway pressure, treatment adherence, diabetes-related outcomes

## Abstract

**Background/Objectives:** There are controversies regarding the effect of obstructive sleep apnea (OSA) treatment with positive airway pressure (PAP) on diabetes-related outcomes. Therefore, we aimed to explore the association of PAP adherence with diabetes-related outcomes in patients with type 2 diabetes mellitus (T2DM) and OSA. **Methods:** In this prospective study, we included T2DM patients diagnosed with OSA during an 8-year period (2015–2023). PAP adherence (optimal usage for > 6 h/night for ≥ 70% of nights), hemoglobin A1c (HbA1c), body mass index (BMI), systolic (SBP) and diastolic blood pressure (DBP), daytime sleepiness (Epworth Sleepiness Scale—ESS) and cardiovascular (CVD) events were recorded. Comparisons of the change in diabetes-related outcomes (follow-up to baseline) in the adherent and non-adherent groups were assessed by analysis of covariance to adjust for relevant confounders. **Results:** Of the 355 patients included, 199 (56%) were PAP adherent. The mean follow-up period was 5.5 years. At the end of the follow up period, the PAP adherent group achieved a greater decrease in HbA1c levels (−1.4 vs. −0.3), SBP (−10.1 vs. −5.5), DBP (−2.9 vs. −0.55) and ESS (−5.9 vs. −4.2) compared to the non-adherent groups. **Conclusions:** Achieving optimal PAP adherence in patients with T2DM and OSA was associated with improved diabetes-related outcomes. Therefore, current practices need to be modified to incorporate systematic assessment and treatment of OSA in these patients.

## 1. Introduction

Obstructive sleep apnea (OSA) is widely acknowledged as one of the most prevalent chronic respiratory disorders in adults characterized by recurrent episodes of complete or partial obstruction of the upper airway during sleep, resulting in intermittent hypoxemia, fragmented sleep, increased oxidative stress, and systemic inflammation [1]. The high prevalence of OSA has significant implications, especially regarding the development and advancement of chronic diseases [2]. Indeed, OSA has been associated with many cardiometabolic comorbidities, including arterial hypertension, insulin resistance, new-onset diabetes, and the likelihood of developing cardiovascular disease (CVD) [3,4,5,6]. Among the various comorbidities linked with OSA, type 2 diabetes mellitus (T2DM) stands out due to its potential to worsen health complications and impede successful treatment [7,8]. A previous meta-analysis of various cohort studies involving more than 60,000 patients with OSA suggested that having OSA can have a similar effect on the development of T2DM as common risk factors like obesity, genetic predisposition, and lack of exercise [9].

Prior research indicates a greater prevalence of OSA in adults with T2DM in comparison to the general population (40–86% versus 9–38%, respectively) [10,11,12,13,14]. Considering the significant cardiovascular risks associated with both OSA and T2DM independently, it is conceivable that the presence of both conditions concurrently may lead to an increased cardiovascular risk beyond that of each condition alone [15]. Previous studies show that patients with T2DM and comorbid OSA are more likely to experience complications such as coronary artery disease, stroke, and microvascular disorders like diabetic nephropathy, retinopathy, and neuropathy, when compared to those without OSA [16,17,18,19,20].

Given that OSA is associated with an increased risk of diabetes-related complications, it is plausible that OSA treatment could have a favorable impact on these complications. Positive airway pressure (PAP) is the treatment of choice for patients with OSA and plays a critical role in improving sleep quality and mitigating associated health risks [21]. However, there are controversies regarding the effect of OSA treatment with PAP on diabetes-related outcomes [6,22,23,24,25], probably due to differences in study design, short follow up and low PAP adherence [6]. More specifically, PAP therapy appears to be effective in improving hemoglobin A1c (HbA1c) levels and thereby improving long-term glycemic control in patients with co-existing OSA and T2DM, although the extent of improvement is linked to the hours of PAP usage [22]. However, no relevant effect was noted on FBG, HOMA-IR or fructosamine levels. Furthermore, the use of PAP in T2DM may have advantages in controlling blood pressure and decreasing diabetic vascular complications such as nephropathy and retinopathy [26,27,28,29,30]. Nevertheless, there is a shortage of long-term data in a larger patient population, specifically focusing on objective PAP adherence data. Therefore, the aim of our study was to explore the association of PAP adherence with clinical outcomes in patients with T2DM and OSA.

## 2. Materials and Methods

### 2.1. Study Patients

We conducted a prospective long-term follow up study for subjects evaluated for suspected OSA in the Sleep Disorders Center, Department of Respiratory Medicine, School of Medicine, University of Crete, in Greece during an 8-year period (2015–2023). The inclusion criteria were (1) age > 18 years, (2) newly diagnosed moderate to severe OSA (apnea–hypopnea index (AHI) ≥ 15 events/h) through an attended overnight polysomnography according to standard criteria, (3) eligible for PAP treatment with a follow-up in-laboratory PAP titration to establish the appropriate PAP settings, and (4) a physician diagnosis of T2DM. The exclusion criteria were refusal to participate, central sleep apnea syndromes, restrictive ventilator syndromes, severe congestive heart failure, a history of life-threatening arrhythmias, severe cardiomyopathy, long-term oxygen therapy, family or personal history of mental illness, drug or alcohol abuse, severe cognitive impairment, history of narcolepsy, or restless leg syndrome. All participants provided written informed consent, and ethical approval was provided by the University Hospital Ethics Committee (approval number: 7370/04-06-2014).

### 2.2. Data Collection

All patients underwent a detailed evaluation that included anthropometric parameters, including body mass index (BMI), medical and sleep history, associated conditions and comorbidities, including physician-based diagnosis for depression, smoking history, and alcohol intake. In addition, we performed overnight attended polysomnography (PSG). Subjective daytime sleepiness, reflected by the Epworth Sleepiness Scale (ESS) was also recorded. The primary outcomes were changes in HbA1c, an estimate of glycemic control, BMI, arterial blood pressure measurements and cardiovascular disease incidents over the follow up period.

#### Epworth Sleepiness Scale

The ESS is currently the most widely used self-reported tool for evaluating daytime sleepiness in clinical environments. The questionnaire is easy to complete and includes eight items for self-assessment. Its aim is to determine the likelihood of falling asleep in eight situations commonly faced. Scoring 10 or higher indicates excessive daytime sleepiness [31].

### 2.3. Polysomnography

All patients underwent a single-night full diagnostic PSG study (Alice 5 Diagnostics System; Respironics, Murrysville, PA, USA) according to standard techniques, with monitoring of the electroencephalogram (using 3 electroencephalogram derivations: frontal, central, and occipital), electro-oculogram, electromyogram, flow (by oronasal thermistor and nasal air pressure transducer), thoracic and abdominal respiratory effort (by respiratory inductance plethysmography), pulse oximetry (SpO_2_), and body position monitoring. Snoring was recorded by a microphone placed on the anterior neck. The definition of apnea and hypopnea followed the American Academy of Sleep Medicine standard criteria [32]. The AHI, calculated as the number of apnea and hypopnea events per hour of sleep, was used to diagnose OSA and assess its severity. OSA was considered mild at AHI of 5 to <15 events/h, moderate at AHI of 15 to <30 events/h, and severe at AHI ≥ 30 events/h. In our study, we only included patients with an AHI ≥ 15/h.

#### 2.3.1. Follow Up

Before starting treatment, every patient visited our PAP clinic for personalized counseling and education on how to use and care for their PAP equipment. Following the initiation of PAP, patients underwent evaluations at the PAP clinic monthly for the first year and then every six months. At these appointments, a clinical evaluation was conducted, and patients were also advised to continue using the device. Immediate action was taken by the nurse at the PAP clinic to address any concerns or questions that could affect compliance negatively. Adjustments to the PAP setting, nose/face mask, or circuit were made after consulting with the sleep physician, if needed. Additionally, patients could reach sleep nurses for treatment consultations through a 24 h telephone line. If there were any doubts about a patient’s willingness to stick to the program, the referring physician would make direct contact with the patient either by phone or through in-person interviews to address any obstacles to consistent compliance. Residual symptoms, such as sleepiness, or any modification in the patient’s overall health status, were documented by the sleep nurse and sleep physician at every follow-up visit.

#### 2.3.2. PAP Adherence

The PAP usage data contained information on mask type (nasal or full face), number of nights using PAP, average nightly usage duration (hours), air leakage, and delivered air pressure. Data on compliance and effectiveness, obtained from the patient’s device, were sent to the lab by the manufacturer or through wireless communication with a cloud-based data reporting tool [33,34]. Monitoring of PAP usage, effective pressures, and residual apnea–hypopnea index occurred at 1, 6, and 12 months after treatment initiation. To improve PAP adherence, unscheduled visits were promptly scheduled for cases of low adherence to PAP therapy.

Meeting the criteria for regular PAP compliance involved using the therapy for an average of 4 h per night on at least 70% of nights [35]. However, to achieve optimal PAP adherence, we set a threshold of 6 h of PAP usage for our analysis [36,37]. Using a threshold of 6 h of documented PAP use per night during the entire study, patients were classified into two groups: those who were PAP adherent (over 6 h/night) and those who were PAP non-adherent (6 h/night or less).

### 2.4. Statistical Analysis

A pilot study was conducted with 50 individuals to determine the sample size. With the data obtained from the pilot study, the sample size was determined as at least 82 individuals to obtain at least 80% power to detect a significant difference in outcome variables between the adherent and non-adherent groups, allowing for a type-I error rate of 0.05. For continuous variables that are normally distributed, results are displayed as mean ± SD; otherwise, they are shown as median (25th–75th percentile). Qualitative variables are displayed in absolute numbers alongside percentages. To compare groups, a two-tailed t-test for independent samples (used with normally distributed data) or a Mann–Whitney U test (for data that are not normally distributed) was employed for continuous variables, along with the chi-square test for categorical variables. Intergroup comparisons of the change in HbA1c (follow-up to baseline) and other outcomes were assessed by analysis of covariance to adjust for age, gender, baseline BMI, smoking status, baseline ESS, and co-morbidities. We used the Kaplan–Meier method and log-rank testing to analyze the association between PAP adherence and cardiovascular events at follow-up. Age was analyzed in two ways: continuously and in categories such as 18–59 and over 60 years. BMI was also evaluated in two ways: continuously and in categories such as under 30 and 30 or more kg/m^2^. The term cardiovascular disease (CVD) in this analysis includes coronary disease, stroke, atrial fibrillation, and heart failure. Results were considered to have significance if *p* values were less than 0.05. Data analysis was conducted using SPSS software (version 25, SPSS Inc., Chicago, IL, USA).

## 3. Results

### 3.1. Patients’ Characteristics

**Figure 1:**
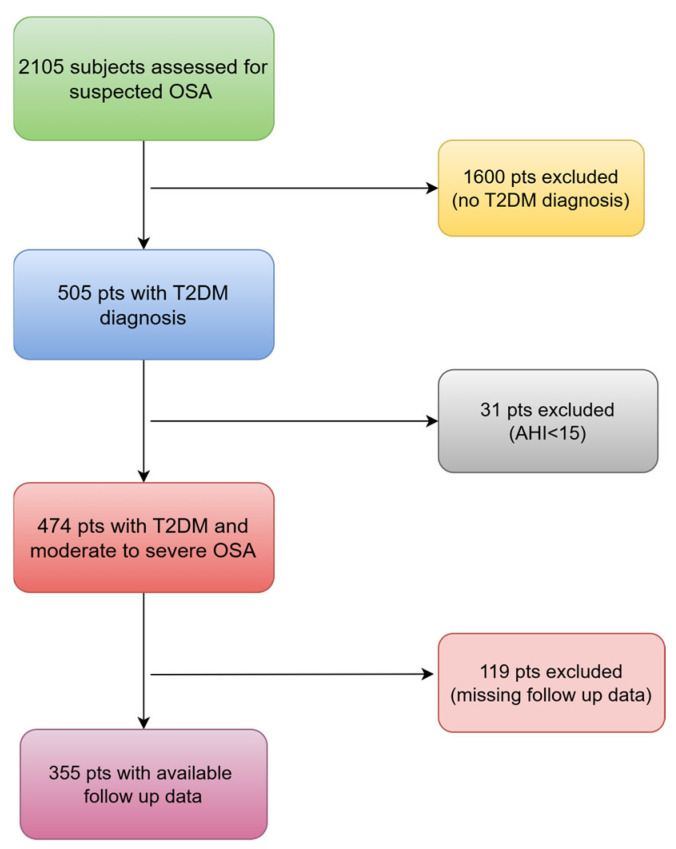
The flowchart of patients that were finally included. OSA: obstructive sleep apnea; T2DM: type 2 diabetes mellitus; pts: patients; AHI: apnea–hypopnea index.

Out of the 2105 adults who were assessed for suspected OSA, 505 of them had T2DM and 474 of them (94%) received a diagnosis of moderate to severe OSA. Among these, 355 patients (75%) with complete follow up data were included in the study (Figure 1). The majority of our patients (84%) used PAP for a minimum of 4 h per night and on at least 70% of nights per week (85%).

Of the 355 patients that started PAP treatment, 199 (56%) had >6 h PAP use per day (PAP adherent group) and 156 (44%) had ≤6 h PAP use per day (PAP non-adherent group). Median PAP adherence was 6.2 (0,14) hours/day. The median hours of PAP use per day were 4.2 (3.3, 5.1) and 7.1 (6.3, 8.2) in the non-adherent and adherent group, respectively. The follow-up period was 5.5 ± 3.8 years. The majority of the participants (97%) were married or had a spouse and had a higher level of education (70%), with no significant differences between non-adherent and adherent groups (98 vs. 97%, *p* = 0.426 and 72 vs. 68%, *p* = 0.833). There were also no differences between the groups regarding the time since T2DM diagnosis, the duration of treatment, the type of treatment (oral medications, insulin, or diet-only), the number of medications used, or the presence of T2DM complications (all *p* > 0.05). The patients’ clinical characteristics are outlined in Table 1.

In the whole sample, the prevalence of co-morbidities ranged from 13% for depression to 69% for hypertension. Almost 13% of patients were taking anti-depressive medications, while almost 59% were taking lipid-lowering therapy. Approximately one-fourth had a history of CVD at baseline. There were no significant differences between those who were PAP adherent and non-adherent in age, gender, BMI, smoking status, baseline blood pressure, HbA1c and comorbidities apart from COPD prevalence, which was higher in the PAP adherent group (32 vs. 19%, *p* = 0.038). At the end of the follow up period, in the PAP group, 3.5% of patients reported changes in anti-diabetic medications (2% vs. 4%, *p* = 0.396).

### 3.2. Effect of PAP Treatment on Diabetes-Related Outcomes

At the end of the follow up period, a significant decrease of 1.1 ± 0.8% in the mean HbA1c level was observed (6.4 ± 1.2% vs. 7.6 ± 1.3%; *p* = 0.001) in the whole group. Furthermore, significant decreases in SBP (124 ± 10 vs. 132 ± 14; *p* < 0.001) and DBP (73 ± 6 vs. 77 ± 11; *p* < 0.001) were noted. There was a nonsignificant BMI reduction in the whole group (36 ± 7 vs. 37 ± 7, *p* = 0.277). However, ESS was significantly improved at follow up (6 ± 4 vs. 12 ± 5; *p* < 0.001). Table 2 summarizes the results for HbA1c levels, blood pressure measurements, ESS and BMI between the groups before and after the follow up period.

The results of the analysis of covariance for diabetes-related outcomes are shown in Table 3. At the end of the follow-up period, the PAP adherent group achieved a greater decrease in HbA1c levels (1.4 vs. −0.3), SBP (−10.1 vs. −5.5), DBP (−2.9 vs. −0.55) and ESS (−5.9 vs. −4.2) compared to the non-adherent groups after adjustments for baseline age, gender, BMI, smoking status, ESS, and co-morbidities.

Twenty-six patients (7.5%) developed CVD and twenty of them (74%) needed hospitalization. Specifically, fifteen (4%) patients developed ACS, three patients stroke (1%), four (1%) AF and four (1%) heart failure. The Kaplan–Meier analysis revealed that patients in the PAP adherence group had a similar event-free survival rate compared to the non-adherent group (log-rank *p* = 0.225).

## 4. Discussion

In this prospective study of patients with T2D and OSA, we aimed to explore if PAP adherence is associated with better diabetes-related outcomes, over a median follow-up of 5.5 years. Our study revealed a significant improvement in glucose metabolism, specifically HbA1C in the group with optimal adherence to PAP treatment. PAP use for at least 6 h per night also improved blood pressure measurements and daytime sleepiness regardless of age, gender, BMI, and presence of comorbidities.

OSA may worsen metabolic abnormalities, including T2DM, but some data suggest that OSA treatment with sufficient adherence could play a protective role [38]. However, patients with OSA and comorbid T2DM seem to have suboptimal adherence to PAP therapy [39,40]. In our study, the median adherence in the whole group was 6.2 h/day, which is relatively high compared to other studies exploring PAP adherence in patients with T2DM [41,42]. Increased adherence could probably be attributed to the comprehensive follow-up process in our sleep lab, which entails frequent visits and personalized counseling and education for patients [36]. This is of particular importance given that increased adherence has the potential to improve diabetes-related outcomes [22].

A key finding of our study is that patients with optimal PAP adherence achieved a decrease in HbA1c levels by 1.4%, compared to 0.3% in the non-adherent group. Patients with T2DM could benefit greatly from this reduction, as a sustained reduction in HbA1c levels by 1% can translate to a clinically meaningful reduction in microvascular complications [22]. Furthermore, the improved glycemic control of our patients does not seem attributable to weight loss, because BMI remained unchanged during the follow-up period. In the literature, the effect of treatment with PAP on markers of glucose metabolism has been conflicting, with available data limited and of varying quality. The variability in PAP adherence could play a significant role in these contradictory results [21]. In line with our findings, some previous studies have highlighted the positive impact of PAP on HbA1c reductions in diabetic patients with OSA [26,43,44,45]. These studies, involving 25 [43] to 1608 patients [44], followed patients for 3 [26,43,45] to 12 months [44], with PAP adherence levels ranging from 4.2 [43] to 5.2 h/night [44]. An important finding from a recent systematic review and meta-analysis of nine RCTs, which involved 807 patients, was the significant decrease in HbA1c levels with PAP treatment in comparison to inactive controls [22]. The study also indicated a direct association between the duration of PAP usage per night and the reduction in HbA1c levels [22]. On the other hand, while other studies have indicated no substantial improvement in HbA1C levels post PAP treatment, these studies had a limited sample size (fewer than 100 patients) and a short follow-up period of less than 1 year [41,42,46,47,48,49]. While the exact mechanisms behind the positive effects of increased hours of PAP use on glycemic control remain unclear in our study, it is probable that they involve reversing some of the ways OSA impacts glucose metabolism [41].

Another important finding of our study was that optimal PAP adherence was associated with significantly lower SBP by 10.1 mm Hg and DBP by 2.9 mm Hg. The decrease in blood pressure observed in our study holds clinical significance, supported by data from the Framingham Heart Study which showed that a 2 mm Hg drop in DBP corresponded to a 15% decrease in cerebrovascular events and transient ischemic attacks [50]. The magnitude of the difference in blood pressure in our study is comparable [26,28,51] or higher [42,48,52] than that found in previous studies on PAP treatment in patients with OSA and comorbid T2DM. Our findings support a dose–response association between higher PAP adherence and reduced blood pressure, emphasizing the significance of PAP adherence in patients with T2DM.

Our study has further substantiated the favorable outcome of PAP on daytime sleepiness. Both groups achieved a comparable reduction in ESS score, with a trend in favor of the patients with optimal PAP adherence (−5.9 vs. −4.2). This result aligns with prior research conducted on individuals with Type 2 diabetes [46]. The potential triple benefit of PAP treatment in addressing daytime sleepiness, HbA1C, and blood pressure may have a positive impact on millions of individuals across the world with diabetes and comorbid OSA. This effect may also plausibly clarify the lower incidence of CVD in PAP adherent patients compared to non-adherent individuals, even though this was statistically insignificant due to the limited number of CVD cases. Nevertheless, further research with extended follow-up periods may provide more insight into this subject.

As adherence to PAP in patients diagnosed with T2DM and OSA plays a critical role in achieving a range of positive clinical outcomes, understanding its significance and addressing barriers with patient education, comfort improvements, and supportive healthcare strategies is crucial for optimizing treatment outcomes. Ongoing research to improve PAP adherence and understanding its implications on managing comorbid diseases is vital for improving the well-being of this at-risk group. Using successful strategies in collaboration with primary care practices to enhance adherence, healthcare providers can support patients in improving their overall health and quality of life.

Our study has some limitations that deserve comment. Firstly, while adjustments were made for various potential confounding factors, residual confounding due to unmeasured variables, including dietary intake and physical activity levels, remains possible. Secondly, the improved PAP adherence observed might be linked to better medication and lifestyle adherence in these individuals, potentially affecting the interpretation of our results. Thirdly, an unexpected reduction in patient follow-up (119 out of 474) was observed due to the impact of the COVID-19 pandemic and the temporary operational constraints of the sleep laboratory in that period. Lastly, as this was a single-center study, involving patients from southern Greece, its findings might not be representative of the entire Greek population.

## 5. Conclusions

The findings of our study suggest that achieving optimal PAP adherence in patients with T2DM and OSA was associated with significantly lower HbA1C, blood pressure, and daytime sleepiness levels. Therefore, it is crucial to further evaluate the role of OSA in managing T2DM, and current practices need to be adjusted to incorporate systematic assessment and treatment of OSA.

## Figures and Tables

**Table 1:** Baseline characteristics of the study population.

Characteristics	Total Population (*n* = 355)	PAP Non-Adherent Group (≤6 h/Night) (*n* = 156)	PAP Adherent Group (>6 h/Night) (*n* = 199)	*p* Value
**Demographics**				
Gender (males)	260 (73%)	115 (74%)	145 (73%)	0.888
Age	62 ± 11	61 ± 11	63 ± 11	0.160
Age ≥ 60 years	209 (59%)	90 (58%)	119 (60%)	0.830
BMI	37 ± 7	37 ± 8	37 ± 7	0.465
BMI ≥ 30	306 (86%)	131 (84%)	175 (88%)	0.373
Current/Former Smoking	(72%)	(73%)	(71%)	0.199
**Symptoms**				
ESS	12 ± 5	12 ± 5	11 ± 5	0.657
ESS ≥ 11	236 (66%)	103 (66%)	133 (67%)	0.908
**Hemoglobin A1c, %**	7.6 ± 1.3	7.5 ± 1.2	7.6 ± 1.1	0.676
**Systolic BP**	132 ± 14	130 ± 12	133 ± 15	0.176
**Diastolic BP**	77 ± 11	77 ± 10	78 ± 11	0.773
**Co-morbidities**				
Hypertension	247 (69%)	112 (72%)	135 (68%)	0.541
CVD	92 (26%)	43 (28%)	49 (25%)	0.614
COPD	94 (26%)	30 (19%)	64 (32%)	0.038
Depression (on medications)	48 (13%)	22 (14%)	26 (13%)	0.831
Hyperlipidemia	208 (59%)	95 (61%)	113 (57%)	0.512
**OSA severity indices**				
AHI	53 ± 23	51 ± 23	54 ± 23	0.376
ODI	56 ± 25	56 ± 25	57 ± 24	0.622
Mean SpO_2_	90 ± 3	90 ± 3	90 ± 3	0.141
Lowest SpO_2_	76 ± 8	76 ± 8	75 ± 8	0.317
TST90	113 ± 76	107 ± 79	119 ± 75	0.268
Follow up (years)	5.5 ± 3.8	5.3 ± 3.2	5.8 ± 2.9	0.189

Data are presented as *n* (%) for categorical variables and mean values ± SD or median (25th–75th percentile) for continuous variables. BP: blood Pressure; BMI: body mass index; PAP: positive airway pressure; ESS: Epworth Sleepiness Scale; CVD: cardiovascular disease; COPD: Chronic Obstructive Pulmonary Disease; AHI: apnea–hypopnea index; ODI: oxygen desaturation index; SpO2: resting room air pulse oximetry; TST90: sleep time with oxygen saturation < 90%.

**Table 2:** Comparison of diabetes-related outcomes between groups before and after follow up period.

	Total Population (*n* = 355)		*p*-Value	PAP Non-Adherent Group (≤6 h/Night) (*n* = 156)		*p*-Value	PAP Adherent Group (>6 h/Night) (*n* = 199)		*p* Value	*p* Value Between Two Groups	
				Baseline	Follow up	0.03	Baseline	Follow up		Baseline	Follow up
Hemoglobin A1c, %	7.6 ± 1.3	6.4 ± 1.2	0.001	7.5 ± 1.2	7.1 ± 1.0		7.6 ± 1.1	6.3 ± 0.3	<0.001	0.676	0.04
Systolic BP	132 ± 14	124 ± 10	<0.001	130 ± 12	123 ± 10	<0.001	133 ± 15	124 ± 3	<0.001	0.176	0.237
Diastolic BP	77 ± 11	73 ± 6	<0.001	77 ± 10	74 ± 8	0.003	78 ± 11	73 ± 7	0.004	0.773	0.321
BMI	37 ± 7	36 ± 7	0.277	37 ± 8	37 ± 8	0.093	37 ± 7	36 ± 6	0.069	0.465	0.516
ESS	12 ± 5	6 ± 4	<0.001	12 ± 5	6.5 ± 4	<0.001	11 ± 5	6 ± 4	<0.001	0.657	0.557

BMI: body mass index; BP: blood pressure; ESS: Epworth Sleepiness Scale; PAP: positive airway pressure.

**Table 3:** Comparison of diabetes-related outcomes between groups before and after follow-up period.

	PAP Non-Adherent Group (≤6 h/Night) (*n* = 156)		*p*-Value	PAP Adherent Group (>6 h/Night) (*n* = 199)		*p*-Value
	Difference (95% CI)	Adjusted Difference (95% CI) *		Difference (95% CI)	Adjusted Difference (95% CI) *	
Hemoglobin A1c, %	−0.4 (0.01, 0.6)	−0.3 (0.01, 0.5)	0.08	−1.3 (0.04, 1.9)	−1.4 (0.04, 2.0)	<0.001
Systolic BP	−6.8 (3.8, 9.7)	−5.5 (0.86, 10,3)	<0.001	−10.4 (7.2, 13.6)	−10.1 (4.9, 15.36)	<0.001
Diastolic BP	−3.3 (1.1, 5.4)	−0.55 (−4, 2.9)	0.003	−4.9 (2.4, 7.3)	−2.9 (0.8, 6.6)	<0.001
BMI	+0.57 (0.03–1.1)	+0.65 (1.5, 2.0)	0.054	+0.9 (0.1, 1.9)	+0.51 (1.0, 20)	0.059
ESS	−5.5 (4.1, 6.9)	−4.2 (2.5, 5.9)	<0.001	−5.5 (4, 5, 6.6)	−5.9 (4.3, 7.4)	<0.001

* Adjusted for baseline age, gender, BMI, smoking status, ESS, and co-morbidities.; BMI: body mass index; BP: systolic blood pressure; ESS: Epworth Sleepiness Scale; PAP: positive airway pressure.

## Data Availability

Data are available upon request.
